# Controllable Skyrmionic Phase Transition between Néel Skyrmions and Bloch Skyrmionic Bubbles in van der Waals Ferromagnet Fe_3‐δ_GeTe_2_

**DOI:** 10.1002/advs.202303443

**Published:** 2023-07-28

**Authors:** Chen Liu, Jiawei Jiang, Chenhui Zhang, Qingping Wang, Huai Zhang, Dongxing Zheng, Yan Li, Yinchang Ma, Hanin Algaidi, Xingsen Gao, Zhipeng Hou, Wenbo Mi, Jun‐ming Liu, Ziqiang Qiu, Xixiang Zhang

**Affiliations:** ^1^ Physical Science and Engineering Division (PSE) King Abdullah University of Science and Technology (KAUST) Thuwal 23955‐6900 Saudi Arabia; ^2^ Tianjin Key Laboratory of Low‐Dimensional Materials Physics and Preparation Technology, School of Science Tianjin University Tianjin 300354 China; ^3^ College of Electronic Information and Automation Aba Teachers University Pixian Street Sichuan 623002 China; ^4^ Guangdong Provincial Key Laboratory of Optical Information Materials and Technology & Institute for Advanced Materials South China Academy of Advanced Optoelectronics South China Normal University Guangzhou 510006 China; ^5^ Laboratory of Solid State Microstructures and Innovation Center of Advanced Microstructures Nanjing University Nanjing 211102 China; ^6^ Department of Physics University of California at Berkeley Berkeley CA 94720 USA

**Keywords:** 2D ferromagnet Fe_3‐δ_GeTe_2_, Dzyaloshinskii–Moriya interaction, Fe atom displacement, magnetic skyrmions

## Abstract

The van der Waals (vdW) ferromagnet Fe_3‐δ_GeTe_2_ has garnered significant research interest as a platform for skyrmionic spin configurations, that is, skyrmions and skyrmionic bubbles. However, despite extensive efforts, the origin of the Dzyaloshinskii–Moriya interaction (DMI) in Fe_3‐δ_GeTe_2_ remains elusive, making it challenging to acquire these skyrmionic phases in a controlled manner. In this study, it is demonstrated that the Fe content in Fe_3‐δ_GeTe_2_ has a profound effect on the crystal structure, DMI, and skyrmionic phase. For the first time, a marked increase in Fe atom displacement with decreasing Fe content is observed, transforming the original centrosymmetric crystal structure into a non‐centrosymmetric symmetry, leading to a considerable DMI. Additionally, by varying the Fe content and sample thickness, a controllable transition between Néel‐type skyrmions and Bloch‐type skyrmionic bubbles is achieved, governed by a delicate interplay between dipole–dipole interaction and the DMI. The findings offer novel insights into the variable skyrmionic phases in Fe_3‐δ_GeTe_2_ and provide the impetus for developing vdW ferromagnet‐based spintronic devices.

## Graphical abstract

A considerable Dzyaloshinskii–Moriya interaction (DMI) induced by Fe atom displacement in 2D ferromagnets Fe_3‐δ_GeTe_2_ is observed for the first time. Furthermore, a controllable transition between Néel‐type skyrmions and Bloch‐type skyrmionic bubbles governed by a delicate interplay between dipole–dipole interaction and the DMI is achieved by tuning the Fe content and sample thickness.

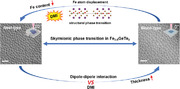

## 1 Introduction

As topological swirling spin textures, skyrmionic spin configurations (i.e., skyrmions and skyrmionic bubbles) have experienced a booming development over the past decade due to their distinctive physical properties and potential applications in high‐speed, low‐dissipation memory, and logic devices.^[^ ^1^, ^2^ ^]^ Skyrmions typically exist in non‐centrosymmetric chiral helimagnets^[^ ^3^, ^4^, ^5^ ^]^ and heavy metal/ferromagnet multilayers.^[^ ^6^, ^7^, ^8^ ^]^ In these material systems, the broken inversion symmetry induces the Dzyaloshinskii–Moriya interaction (DMI),^[^ ^9^, ^10^ ^]^ which generates either Bloch‐ or Néel‐type skyrmions depending on the direction of symmetry breaking. By contrast, skyrmionic bubbles are generally hosted by a delicate interplay between dipole–dipole interaction and uniaxial anisotropy in centrosymmetric ferromagnets.^[^ ^11^, ^12^, ^13^ ^]^ From a topological point of view, skyrmionic bubbles and Bloch‐type skyrmions share the same topological classification.^[^ ^14^ ^]^ However, due to the lack of the DMI, skyrmionic bubbles possess two additional degrees of freedom, namely, helicity and vorticity, which endows them with a unique set of properties including switchable helicity and multiple topologies.^[^ ^1^ ^]^ For practical applications, different skyrmionic spin configurations are expected to achieve specific functionalities based on their distinct characteristics. For instance, the switchable helicity in skyrmionic bubbles could act as binary data bits,^[^ ^15^, ^16^ ^]^ while skyrmions might be more suitable for the racetrack memory because their fixed helicity guarantees a consistent motion direction when driven by a spin‐polarized current.^[^ ^2^ ^]^ Thus, before designing spintronic devices based on a topological magnetic system, it is essential to determine the system's skyrmionic phase.

**Figure 1:**
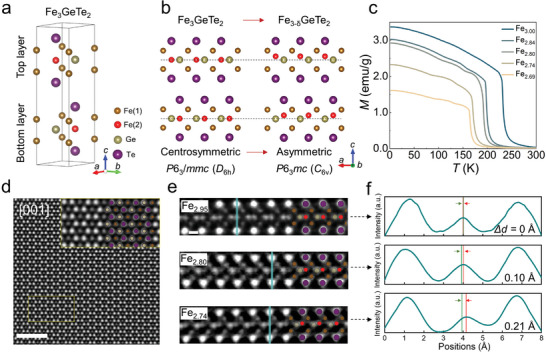
Structural and magnetic properties of Fe_3‐δ_GeTe_2_ crystals. a) Crystal structure model of Fe_3_GeTe_2_. b) Schematic of atom arrangements of Fe_3‐δ_GeTe_2_ viewed from *ac*‐plane with Fe(2) atom displacement along *c*‐axis and structure symmetry decreased from *P*6_3_/mmc to *P*6_3_mc. c) *M*–*T* curves measured under a perpendicular magnetic field of 10 mT for Fe_3‐δ_GeTe_2_ with different Fe contents. d) The HAADF‐STEM image viewed along the [001] orientation. The inset shows the magnified image based on the yellow rectangular region overlapped with the schematic of the atomic structure. Scale bar: 2 nm. e) The HAADF‐STEM images observed along the [100] orientation of Fe_3‐δ_GeTe_2_ lamellas: Fe_3.00_GeTe_2_, Fe_2.80_GeTe_2_, and Fe_2.74_GeTe_2_, respectively. Scale bar: 2 Å. f) Line profiles of the STEM intensity shown in (e). The green line represents the central position between two Te atoms and the red line represents the center of the Fe(2) atom. The distance between the red and green lines indicates the Fe(2) atom displacement Δ*d* along the *c*‐axis.

In recent years, research on skyrmionic spin configurations has expanded from traditional magnetic materials to van der Waals (vdW) ferromagnets such as Cr_2_Ge_2_Te_6_,^[^ ^17^ ^]^ Fe*_n_* GeTe_2_ (*n* = 3, 5),^[^ ^18^, ^19^, ^20^, ^21^, ^22^, ^23^, ^24^, ^25^, ^26^, ^27^, ^28^ ^]^ and (Fe_0.5_Co_0.5_)_5_GeTe_2_.^[^ ^29^ ^]^ Since 2D vdW materials exhibit great advantages over traditional materials systems (e.g., high flexibility, atomic thickness stability, and LEGO‐like modularity), their combination with skyrmionic spin configurations may provide a more spacious platform for the future development of spintronics. Among the various vdW ferromagnets with topological magnetism, Fe_3_GeTe_2_ has attracted the most attention because of its relatively high Curie temperature (*T*_C_), metallic conductivity, and robust perpendicular magnetic anisotropy (PMA).^[^ ^30^ ^]^ It has widely been considered that Fe_3_GeTe_2_ possesses a centrosymmetric hexagonal crystal structure with an AB‐stacked Fe_3_GeTe_2_ bilayer,^[^ ^31^, ^32^, ^33^ ^]^ as schematically illustrated in **Figure** **1**a. The inversion symmetry rules out the DMI, and thus, it is not strange to observe skyrmionic bubbles at an appropriate magnetic field and temperature range.^[^ ^18^ ^]^ Surprisingly, however, recent studies have demonstrated the emergence of Néel‐type skyrmions in Fe_3_GeTe_2_.^[^ ^22^ ^]^ As Fe_3_GeTe_2_ nanoflakes may slowly degrade in ambient conditions, studies have proposed that the interface between their pristine and oxidized surfaces has a considerable interface DMI.^[^ ^22^, ^23^ ^]^ Nevertheless, it is worth mentioning that Néel‐type skyrmions were also observed through scanning tunneling microscopy (STM) in Fe_3_GeTe_2_ flakes cleaved in situ under an ultrahigh vacuum,^[^ ^34^ ^]^ indicating that the oxidized interface should not be the dominant factor—or at least the only factor—for the emerging Néel‐type skyrmions. Thus, the underlying physical mechanism responsible for the emergence of the DMI and the observed variable skyrmionic phases in Fe_3_GeTe_2_ remains elusive despite the numerous efforts devoted to uncovering it.^[^ ^21^, ^22^, ^35^, ^36^, ^37^ ^]^

It was experimentally reported that the as‐prepared Fe_3_GeTe_2_ crystals generally exhibit a slight deficiency in Fe concentration, which significantly impacts the material's intrinsic magnetic properties^[^ ^38^ ^]^ including *T*_C_, net magnetization, and magnetic anisotropy. For instance, even a small Fe deficiency of 10% can result in a drastic reduction in *T*_C_ of more than 60 K. However, despite the remarkable effect, previous studies have overlooked the influence of Fe content on the crystal structure and skyrmionic phase of Fe_3_GeTe_2_.^[^ ^31^, ^32^, ^38^ ^]^ Therefore, in the present study, we conducted a systematic investigation of the magnetic and structural properties of Fe‐deficient Fe_3‐δ_GeTe_2_ (*δ* represents the variation of Fe content). Using high‐angle annular dark‐field scanning transmission electron microscopy (HAADF‐STEM), we observed a significant displacement of Fe(2) atoms along the *c*‐axis (as schematically illustrated in Figure 1b) and the offset increased with the reduction of Fe content. First‐principles calculations demonstrated that the Fe(2) displacement transformed the original centrosymmetric structure with *P*6_3_/mmc space group into the non‐centrosymmetric structure with *P*6_3_mc space group, inducing an effective DMI with its strength proportional to the atomic displacement. By delicately tuning the Fe content and sample thickness, we further achieved a controllable skyrmionic phase transition from Néel‐type skyrmions to Bloch‐type skyrmionic bubbles. Micromagnetic simulations indicated that the transition arose from the competition between the DMI and dipole–dipole interaction. Our findings may resolve the protracted debate on both the origin of the DMI and variable skyrmionic phases in Fe_3‐δ_GeTe_2_, which is of great importance to practical applications of skyrmionic spin configurations in the vdW ferromagnets.

## 2 Results and Discussion

### 2.1 Magnetic Properties and Crystal Structure of Fe_3‐δ_GeTe_2_ Crystals

First, we synthesized a series of Fe_3‐δ_GeTe_2_ single crystals with various Fe contents (*δ* = 0, 0.16, 0.20, 0.26, and 0.31) using the chemical vapor transport (CVT) method. Details about the crystal growth and composition determination are described in the Experimental Section and Figure S1, Supporting Information, respectively. Magnetic characterization of the Fe_3‐δ_GeTe_2_ crystals was performed with the external magnetic field (*H*) perpendicular to the *ab*‐plane. Figure 1c presents the temperature‐dependent magnetization (*M*–*T*) curves measured in the field‐cooled (FC) protocol under a small *H* of 10 mT. By taking their first derivative (d*M/*d*T*), we were able to determine the *T*_C_ of the Fe_3‐δ_GeTe_2_ crystals, as depicted in Figure S2, Supporting Information. The results demonstrated that their *T*_C_ fell within the temperature range of 165–233 K and increased monotonically as the Fe content increased. These features are quantitatively consistent with those found in previous studies,^[^ ^31^, ^32^, ^38^ ^]^ suggesting the reliability of our crystal quality.

Subsequently, we performed HAADF‐STEM to analyze the influence of the Fe content on the atomic arrangement in Fe_3‐δ_GeTe_2_ (Figure 1d–f). Crystals with three typical Fe content (i.e., Fe_3.00_GeTe_2_, Fe_2.80_GeTe_2_, and Fe_2.74_GeTe_2_) were chosen for the detailed studies. To understand the spatial atomic arrangement, both the [001]‐ and [100]‐oriented lamellae (thickness is fixed to be ≈100 nm) were fabricated from all three crystals using a focused ion beam (FIB) (see Experimental Section for fabrication details). Figure 1d presents a typical HAADF‐STEM image of the [001]‐oriented Fe_2.80_GeTe_2_ lamella. In the image, the gray and bright spots represent the Fe(1) and Te‐Fe(2)‐Te‐Ge atomic columns in which the variation in brightness reflects their respective average atomic numbers (see the inset for a magnified image of the yellow‐rectangular region). As depicted in Figure 1d, the Fe(1) atom columns (marked with brown circles) were hexagonally surrounded by six Te‐Fe(2)‐Te‐Ge atomic columns (marked with purple and pale green circles), which was highly consistent with the top view of the conventional Fe_3_GeTe_2_'s crystal structure (Figure 1a). Similar hexagonal atomic arrangements were also observed in the [001]‐oriented Fe_3.00_GeTe_2_ and Fe_2.74_GeTe_2_ lamellae (Figure S3, Supporting Information). These observations demonstrate that the variation in Fe content did not cause noticeable atomic displacement in the *ab* plane. By contrast, a significant atomic displacement of the Fe(2) atoms was observed in the [100]‐oriented lamella. Figure 1e presents the HAADF‐STEM images recording the atomic arrangement of the top layer of the Fe_3‐δ_GeTe_2_ unit cell. For Fe_3.00_GeTe_2_ (top panel of Figure 1e), the Fe(2)‐Ge slabs (marked with the red and pale green circles) were sandwiched between two Te layers (marked with the purple circles), and the Fe(2) and Ge atoms were in one horizontal plane. However, in Fe_2.80_GeTe_2_ and Fe_2.74_GeTe_2_ (middle and lower panels of Figure 1e), the Fe(2) atoms deviated from the Ge plane along the *c*‐axis, and the offset (*Δd*) increased as the Fe content decreased, as verified by the STEM intensity profile in Figure 1f. This deviation was also observed for the Fe(2) atoms in the bottom layer of the Fe_3‐δ_GeTe_2_ unit cell, and both their offset and displacement directions were the same as those in the top layer (Figure S4, Supporting Information). Based on the HAADF‐STEM observations along different crystal axes, we can conclude that in Fe‐insufficient Fe_3‐δ_GeTe_2_ the Fe(2) displacement only occurs along the *c*‐axis, and other atoms are not shifted. Owing to the Fe(2) atom displacement, the space group of Fe_2.80_GeTe_2_ and Fe_2.74_GeTe_2_ was not a centrosymmetric *P*6_3_/mmc structure anymore but had rather transformed into the *P*6_3_mc structure. We further simulated the STEM images based on the *P*6_3_mc structure (see Note S1, Supporting Information, for details about the simulations). The simulated images agreed well with our experimental observations (Figure S5, Supporting Information), confirming the reliability of our structural determination.

### 2.2 The Physical Origin of the DMI in Fe_3‐δ_GeTe_2_

**Figure 2:**
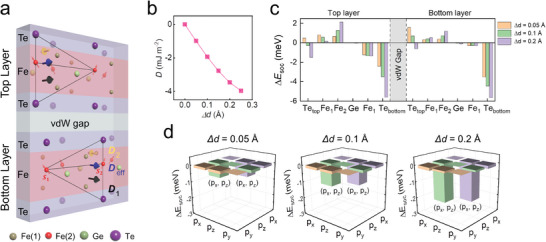
The origin and strength of DMI in Fe_3‐δ_GeTe_2_. a) Schematic of a Fe_3‐δ_GeTe_2_ unit cell. The DMI induced between Fe/top Te atoms(***D***_2_) and between Fe/bottom Te atoms (***D***_1_) are in the opposite direction. With the Fe(2) atom displacement along the *c*‐axis, ***D***_1_ is no longer equal to ***D***_2_ and an effective DMI ***D***_eff_ occurs. b) DFT‐calculated DMI of Fe_3‐δ_GeTe_2_ with varying displacements. The negative *D*_DFT_ represents the clockwise chirality. c) Atomic layer‐resolved Δ*E*_SOC_ related to DMI constant with Fe(2) atom displacement. d) p orbital‐resolved Δ*E*_SOC_ matrix elements of the bottom Te atom, in which (p_x_,p_z_) represent p_x_–p_z_ orbital hybridization of bottom Te atoms.

Because the *P*6_3_mc space group is non‐centrosymmetric, a pronounced DMI was naturally expected in the Fe_2.80_GeTe_2_ and Fe_2.74_GeTe_2_. As a chiral interaction between two neighboring spins, the presence of the DMI requires both broken inversion symmetry in the crystal structure and the strong spin‐orbit coupling (SOC) effect.^[^ ^8^ ^]^ The DMI vector ***D*** can be expressed as follows

(1) $$D=D\times \left(u_{12}\times z\right)$$

where *D* represents the DMI constant that fully determines the magnitude of the DMI; ***u***_12_ represents the unit vector between two neighbor spins; and ***z*** represents the unit vector pointing from the magnetic atom layer to the heavy atom layer, and it is perpendicular to the atomic interface between them. For the non‐centrosymmetric Fe_3‐δ_GeTe_2_, since its inversion symmetry was broken by the Fe(2) atom displacement, the interactions between two neighboring Fe(2) atoms and the adjacent heavy metalloid atom Te could act as the DMI source. As schematically illustrated in **Figure** **2**a, in a Fe_3‐δ_GeTe_2_ unit cell, two DMI sources exist for each vdW layer; that is, the Fe(2)/lower Te interface corresponds to ***D*_1_** (black arrow), and the Fe(2)/upper Te interface corresponds to ***D*_2_** (yellow arrow). According to Equation (1), their direction is established as parallel to the Fe/Te atom interface (magnetic/heavy‐atomic layers) and is in‐plane isotropic. These features correspond to the Fert–Levy DMI,^[^ ^39^ ^]^ which is widely reported in the heavy metal/ferromagnet multilayers and accounts for the Néel‐type skyrmions. Moreover, since ***z*** has opposite directions for ***D*_1_** and ***D*_2_** while ***u*_12_** is identical, their direction is opposite along the Fe/Te atom interface. The effective DMI should be ***D*_eff_ = *D*_1_** − ***D*_2_**. For the centrosymmetric Fe_3_GeTe_2_ (without atom displacement), they cancel each other out since the magnitude of ***D*_1_** is equal to that of ***D*_2_**. However, when Fe(2) atoms are displaced from their initial sites, the two DMI vectors are no longer equal in magnitude, leading to an effective net DMI (***D*_eff_**, blue arrow) in each layer. Since the direction of ***D*_eff_** in the top layer is as same as that in the bottom layer (Figure 2a), the total DMI vector (***D*_total_**) in Fe_3‐δ_GeTe_2_ is regarded as the accumulation of the ***D*_eff_** in each layer. In addition, considering that all the Fe(2) sites show a similar contrast in the HAADF‐STEM image (Figure S4, Supporting Information), the non‐stoichiometric Fe(2) atoms should be uniformly distributed in Fe_3‐δ_GeTe_2_. Moreover, their displacement is almost identical in different layers. Thus, the DMI is considered globally uniform in the material.

Next, based on the abovementioned model, we conducted first‐principles calculations to quantitatively study the relationship between Fe(2) atom displacement and the DMI. Details about the calculations are described in the Experimental Section. The dependence of the calculated DMI constant (*D*_DFT_) on the Fe(2) atom offset is depicted in Figure 2b, where the negative *D*_DFT_ indicates a clockwise spin chirality. The absolute value of *D*_DFT_ was observed to increase almost linearly with the increasing Fe(2) atom offset. Moreover, our structural analysis indicated that the Fe(2) atom offset increases with the reduction of Fe content (Figure 1e). Thus, we concluded that a lower Fe content corresponds to a stronger DMI in the Fe_3‐δ_GeTe_2_. In addition to the symmetry breaking of the crystal structure, SOC is also indispensable for the emergence of the DMI. To elucidate the respective DMI contributions of individual atoms in the Fe_3‐δ_GeTe_2_ unit cell, we calculated the atom‐resolved SOC energy differences (Δ*E*_SOC_), which is proportional to the DMI constant (see Experimental Section for details about their relationship). Figure 2c shows the Δ*E*_SOC_ at different atom sites and their dependence on the Fe(2) atom offset. Because *D*_DFT_ is negative in Fe_3‐δ_GeTe_2_ (Figure 2b), the negative Δ*E*_SOC_ values represented a positive contribution to the effective DMI. We observed that the Δ*E*_SOC_ contributed by the bottom Te atoms to be negative and its absolute value to be much larger than that of the other atoms; simultaneously, the absolute value of Δ*E*_SOC_ increased correspondingly with increasing atomic offset, which is similar to the change tendency of *D*_DFT_ with the atomic offset. These results implied that the SOC of the bottom Te atoms plays a dominant role in the emerging DMI. Furthermore, the p orbital‐resolved Δ*E*_SOC_ deduced from SOC matrix elements of the bottom Te atom were calculated. As Figure 2d indicates, the strongest contribution to Δ*E*_SOC_ comes from the matrix element of p_x_ and p_z_ orbitals; that is to say, the p*_x_*–*p_z_* orbital hybridization of the bottom Te atom mainly offered the SOC for the emerging DMI in the Fe_3‐δ_GeTe_2_.

### 2.3 The Variation of the Skyrmionic Phase with the Fe Content

**Figure 3:**
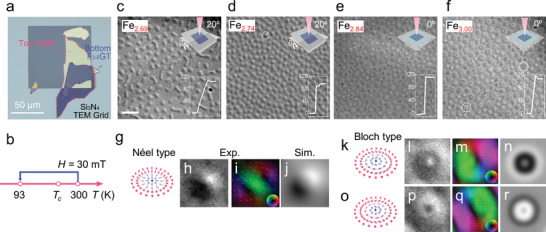
Skyrmionic spin configurations in ≈100 nm thick Fe_3‐δ_GeTe_2_ nanoflakes with various Fe content at 93 K. a) A typical optical microscopy image of h‐BN/Fe_3‐δ_GeTe_2_ heterostructures on the Si_3_N_4_ TEM grid. b) The schematic diagram for FC procedure. c–f) Under‐focused L‐TEM images taken at 93 K after the 30 mT FC process with various Fe concentrations as follows: c) Fe_2.69_GeTe_2_, d) Fe_2.74_GeTe_2_, e) Fe_2.84_GeTe_2_, and f) Fe_3.00_GeTe_2_. The defocused value of images is 3 mm. The insets on the top right are the schematic graphs of sample tilting for L‐TEM imaging. Néel‐type skyrmions were captured with tilting angles *θ* of 20° in (c) and (d) and Bloch‐type chiral bubbles were observed under zero tilt conditions in (e) and (f). Scale bar: 1 µm. The insets on the bottom right are the accurate thicknesses of Fe_3‐δ_GeTe_2_ nanoflakes measured by AFM. h,l,p) The L‐TEM images of Néel type skyrmions and Bloch‐type skyrmions with different chirality, respectively, magnified from (d) and (f). The corresponding magnetic inductions (i,m,q), simulated L‐TEM phase contrasts (j,n,r), and illustrations (g,k,o) are also shown, respectively. “Exp.” and “Sim.” mean “experiment” and “simulation,” respectively.

The above findings demonstrated that a stronger DMI was observed in Fe_3‐δ_GeTe_2_ with a lower Fe content. This suggests that Néel‐type skyrmions are more likely to be formed in Fe‐insufficient Fe_3‐_ *_δ_* GeTe_2_, while dipole skyrmions are more likely to be observed in Fe‐rich Fe_3‐_ *_δ_* GeTe_2_. To investigate the correlation between the Fe content and the skyrmionic phase, we performed Lorentz transmission electron microscopy (L‐TEM) on exfoliated [001]‐oriented Fe_3‐δ_GeTe_2_ (*δ* = 0.00, 0.16, 0.26, and 0.31) flakes (≈100 nm in thickness). The flakes were covered with hexagonal boron nitride (h‐BN) using a dry‐transfer method in a glove box to prevent the oxidation of surface layers during the transfer and measurement process (see Experimental Section). **Figure** **3**a presents a typical optical microscopy image of an h‐BN/Fe_3‐δ_GeTe_2_ heterostructure on a Si_3_N_4_ TEM grid. The electron energy loss spectroscopy (EELS) line scan result (Figure S6, Supporting Information) revealed no detectable oxygen signal in the h‐BN/Fe_3‐δ_GeTe_2_ heterostructure, which excluded the possibility of interfacial oxidation.

For the L‐TEM measurements, the samples were cooled from 300 (well above *T*_C_) to 93 K with an out‐of‐plane magnetic field of 30 mT (as schematically illustrated in Figure 3b). After the FC operation, no magnetic contrast was observed in the L‐TEM images of Fe_3‐_ *_δ_* GeTe_2_ (*δ* = 0.26 and 0.31) when the electron beam was injected along the normal direction of the samples (Figure S7, Supporting Information). By contrast, when the samples were titled at a certain angle (*θ*) away from the *ab*‐plane, dark‐bright domains were observed, as depicted in Figure 3c,d. It is well established that the magnetic contrast observed in an L‐TEM image is generally attributed to the electron beam deflection by the in‐plane magnetic field while passing through the specimen. Under the Fresnel mode of L‐TEM, one can always observe the magnetic contrast of the Bloch‐type domain walls regardless of whether the sample is tilted. By contrast, the Néel‐type domain walls do not display any magnetic contrast without the sample being tilted because the change in intensity produced by the Lorentz force is compensated or canceled at any position.^[^ ^40^ ^]^ The angle‐dependent contrast variation observed in our experiments suggested that the imaged dark‐bright domains were Néel‐type. Figure 3i presents the corresponding in‐plane spin structures of the dark‐bright domain (Figure 3h) obtained through a transport‐of‐intensity equation (TIE) analysis. The green and purple regions represent anti‐parallel in‐plane domains, whereas the dark regions indicate out‐of‐plane domains. The in‐plane magnetic induction of the dark‐bright domains was demonstrated to be composed of a pair of conjoined clockwise and counterclockwise spin swirls, which agrees with the calculated magnetic induction map for the Néel‐type skyrmions. We further simulated the L‐TEM image using the standard spin texture of a Néel‐type skyrmion with titling *θ* of 20°. As shown in Figure 3j, the simulated L‐TEM magnetic contrasts were highly consistent with the experimental observations (Figure 3h), suggesting that the dark‐bright domains observed in the Fe_3‐_ *_δ_* GeTe_2_ (*δ* = 0.26 and 0.31) were Néel‐type skyrmions.

In the case of the Fe_3‐_ *_δ_* GeTe_2_ (*δ* = 0.00 and 0.16), the bubble‐like domains could be imaged without tilting the sample (*θ* = 0°), suggesting that they were Bloch‐type. Magnified images further revealed that their contrast was ring‐like and that some displayed completely inverse contrast variation (Figure 3l,p). Furthermore, TIE analysis revealed that the bubble‐like domains in the Fe_3‐_ *_δ_* GeTe_2_ (*δ* = 0.00 and 0.16) were Bloch‐type skyrmionic bubbles, while the inverse contrast variation implied that their in‐plane spin swirling direction was reversed. These results could be further confirmed by the good consistency between the simulated L‐TEM image based on a standard Bloch‐type skyrmionic bubble and our experimental results, as depicted in Figure 3k–r. Here, we should also note here that the random helicity of skyrmionic bubbles can be attributed to the lack of a strong enough DMI in Fe_3‐_ *_δ_* GeTe_2_ (*δ* = 0.00 and 0.16), which agrees with our first‐principles calculations that the DMI is weak in the higher Fe‐content Fe_3‐δ_GeTe_2_ due to its insignificant Fe(2) atom displacement. Thus, the Bloch‐type skyrmionic bubbles with random helicity in Fe‐rich Fe_3‐δ_GeTe_2_ originate from the delicate balance between the magnetic anisotropy energy and demagnetization energy.

### 2.4 Thickness‐Dependent Skyrmionic Phase Transition

**Figure 4:**
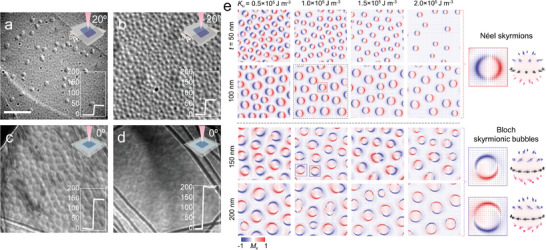
Thickness‐dependent skyrmionic phase transition in Fe‐deficient Fe_2.74_GeTe_2_ nanoflakes at 93K. a–d) Under‐focused L‐TEM images captured from Fe_2.74_GeTe_2_ nanoflakes with thicknesses of 44 (a), 70 (b), 142 (c), and 203 nm (d). The insets at the bottom right are the thickness of Fe_2.74_GeTe_2_ nanoflakes. The illustration at the top right represents the tilted conditions for L‐TEM imaging. Néel‐type skyrmions were observed in the thin Fe_2.74_GeTe_2_ nanoflakes with a 20° tilting angle (a,b). Bloch‐type chiral bubbles existed in the Fe_2.74_GeTe_2_ nanoflakes with thick thicknesses (c,d). The defocused value is 3 mm. Scale bar: 1 µm. e) Skyrmionic phase diagram as a function of thickness *t* and magneto‐crystalline anisotropy constant *K*_u_. The corresponding magnified real space planar skyrmionic configurations and topological presentation of Néel skyrmion and Bloch skyrmionic bubbles with opposite helicity (right panel).

We further found that Bloch‐type skyrmionic bubbles could also appear in the Fe_3‐δ_GeTe_2_ flakes with lower Fe‐content and a sample thickness (*t*) well above 100 nm, despite the existence of a pronounced DMI. **Figure** **4**a–d shows the L‐TEM images of the Fe_2.74_GeTe_2_ flakes with a thickness ranging from 44 to 203 nm at 93 K after the FC procedure. In the 44 and 70 nm‐thick flakes, Néel‐type skyrmions were identified as they did not show magnetic contrast without sample tilting (see Figure S8, Supporting Information). Intriguingly, one can clearly observe a transition from Néel‐type skyrmions to Bloch‐type skyrmionic bubbles when the thickness reached 142 nm (Figure 4c). These results implied that the sample thickness offers another degree of freedom in tuning the skyrmionic phase transition in the Fe_3‐δ_GeTe_2_, in addition to the Fe content. For a vdW ferromagnetic system, sample thickness plays a crucial role in determining its dipole–dipole interaction and uniaxial anisotropy; specifically, increasing the sample thickness generally decreases the PMA while simultaneously enhancing the strength of the dipole–dipole interaction.^[^ ^19^, ^41^ ^]^ To identify their perspective roles in the thickness‐dependent skyrmionic phase transition, micromagnetic simulations were conducted (see Note S2, Supporting Information, for details). First, we simulated the magnetic domain states of the Fe_2.74_GeTe_2_ flakes based on their physical parameters established in the experiments. As indicated in the dashed box in Figure 4e, field‐free Néel‐type skyrmions can be obtained after a simulated FC procedure, which agrees with the experimental results (Figure 3d) as well as confirms the reliability of our model. Next, we varied the magneto‐crystalline anisotropy constant (*K*_u_) in the range of 0.5–2 J m^−3^ and simulated the evolution process of magnetic domain structures for different thicknesses varying from 50 to 200 nm. As shown in Figure 4e, when the sample thickness was fixed, varying *K*_u_ was unable to drive an appreciable transition in the skyrmionic phase at each thickness. However, as the thickness was increased, the strength of the dipole–dipole interaction was enhanced, leading to the spins gradually rotating toward the perpendicular to the radial plane. As a result, a transition from Néel skyrmions to Bloch skyrmionic bubbles was observed. From the simulation results, we deduced that enhancing the dipole–dipole interaction facilitates the formation of Bloch‐type skyrmionic bubbles, and also that the observed thickness‐dependent transition of the skyrmionic phase in Fe_3‐_ *_δ_* GeTe_2_ is primarily governed by the competition between the dipole–dipole interaction and the DMI.

It should be noted that the dry transfer method using polydimethylsiloxane (PDMS) may induce a small strain in the Fe_3‐δ_GeTe_2_ flakes.^[^ ^42^ ^]^ Via magneto‐elastic coupling, the strain can effectively tune the magnetic anisotropy energy and even cause topological phase transitions in ferromagnetic thin films.^[^ ^43^, ^44^ ^]^ Considering that the strain is usually of thickness dependence,^[^ ^45^ ^]^ thus the strain‐induced magnetic anisotropy energy should also vary with the Fe_3‐δ_GeTe_2_ flake thickness. In our micromagnetic simulations, *K*_u_ was tuned in a wide range of 0.5–2 J m^−3^ in each thickness (Figure 4e), whereas the skyrmionic phase keeps unchanged, meaning that the possible strain‐induced *K*_u_ variation could not be the substantial reason for the skyrmionic phase transition. On the other hand, a recent study revealed that a 5% compressive strain could lead to a structural transition of Fe_3_GeTe_2_,^[^ ^46^ ^]^ which might change the DMI strength. While this strain threshold is an order of magnitude larger than the actual strain in PDMS‐transferred flakes (typically less than 0.22%).^[^ ^42^ ^]^ Therefore, the transfer‐process‐induced strain is unlikely to cause the structural transition and change the DMI strength. Based on the above analyses, we believe that the strain effect should not be responsible for the observed thickness‐dependent skyrmionic phase transition.

## 3 Conclusion

In summary, we have systematically studied the crystal structure and magnetic properties of Fe_3‐δ_GeTe_2_, with varying Fe concentrations. By employing HAADF‐STEM, we have observed a significant displacement of Fe(2) atoms along the *c*‐axis in Fe‐insufficient Fe_3‐δ_GeTe_2_, which increased with the decrease of the Fe content. This is the first time that such a displacement has been revealed. First‐principles calculations indicated that the Fe(2) displacement could break the inversion symmetry of the original crystal structure from centrosymmetry (*P*6_3_/mmc) to non‐centrosymmetry (*P*6_3_mc), inducing a considerable DMI with its strength proportional to the atomic displacement. Furthermore, we realized a controllable skyrmionic phase transition from Néel‐type skyrmions to Bloch‐type skyrmionic bubbles by delicately tuning the Fe content and sample thickness. Micromagnetic simulations indicated that the transition mechanism originates from the subtle competition between DMI and dipole–dipole interaction. This study could potentially resolve the persistent debate regarding the origin of the DMI and the variable skyrmionic phase in Fe_3‐δ_GeTe_2_, as well as provide new insights into generating novel spin textures in vdW ferromagnets.

## 4 Experimental Section

### Crystal Synthesis

The Fe_3‐δ_GeTe_2_ crystals were synthesized by the CVT method, which is similar to the author's previous works.^[^ ^25^, ^47^, ^48^ ^]^ To synthesize Fe_3‐δ_GeTe_2_ with different Fe content, high‐purity Fe, Ge, and Te powders (Alfa Aesar) were mixed up in the ratio of 3‐*δ*:1:2, where *δ* is a slight reduction and packed into vacuum‐sealed ampoules together with a small amount of iodine. Then the ampoules were placed in a furnace with a temperature range of 700–750 °C for 1 week. The ampoules cooled to room temperature naturally and were removed from the furnace.

### TEM Lamellas and h‐BN/Fe_3‐δ_GeTe_2_ Preparation

The TEM lamellas were fabricated using the Helios G4 UX FIB system (Thermo Fisher Scientific), equipped with a Ga^+^ beam source. To minimize the irradiation damage to the lamellae, low‐energy (2–5 kV) final polishing was required. The h‐BN/Fe_3‐δ_GeTe_2_ nanoflakes were fabricated by the dry transfer technique. First, the Fe_3‐δ_GeTe_2_ nanoflakes exfoliated by Scotch tapes were transformed onto a PDMS stamp. The nanoflake with proper thickness was then selected and transferred to a Si_3_N_4_ TEM grid (CleanSiN) using a precise transfer platform (Metatest, E1‐M). In the same way, the thin h‐BN nanoflake was stacked onto the Fe_3‐δ_GeTe_2_ nanoflake. The entire process was completed in the glove box, with the oxygen concentration lower than 1 ppm. Then the samples were vacuum‐sealed until future tests.

### Sample Characterization

The EDS results were collected using the Zeiss MERLIN scanning electron microscope (SEM). The atomic resolution HAADF‐STEM images and EELS results were obtained using FEI Titan Themes Cubed G2 300 (Cs Probe) TEM at 300 kV. The nanoflakes' thicknesses were determined by atomic force microscopy (AFM) measurements (Dimension Icon SPM). The magnetization measurements were carried out using the superconducting quantum interference device (SQUID) MPMS3 magnetometer (Quantum Design).

### L‐TEM Imaging

The skyrmions in the nanoflakes were observed using FEI Titan Themes Cubed G2 300 TEM under Lorentz–Fresnel Mode. To achieve in situ observation at variable temperatures (93–363 K), a single‐tilt in situ cooling holder (Gatan Model 613) was used. The objective lens was switched off before the sample was loaded in TEM. The external vertical magnetic field was applied by slowly increasing the Lorentz lens current for the in situ FC process. The magnetic induction maps were processed by QPT software based on the TIE.

### First‐Principles Calculations

The Vienna ab initio simulation package (VASP) was employed to perform structural and magnetic calculations on Fe_3‐δ_GeTe_2_, using the generalized gradient approximation for the exchange and correlation function by Perdew, Burke, and Ernzerhof.^[^ ^49^, ^50^, ^51^ ^]^ The Kohn–Sham single‐particle wave functions were expanded using a plane wave basis set with a kinetic energy cutoff set at 600 eV. The energy and force convergence criteria of 10^−7^ eV and 10^−3^ eV Å^−1^, respectively, were adopted. The DFT‐D3 method was used to include the long‐range correlation when evaluating the vdW interaction.^[^ ^52^ ^]^ A Monkhorst‐Pack 4 × 16 × 1 k‐point grid was adapted to sample the Brillouin zone. The structural calculations of the Fe_3‐δ_GeTe_2_ were executed by inputting both standard Fe_3_GeTe_2_ structure and experimentally measured atomic offset into VASP. The symmetry of the Fe_3‐δ_GeTe_2_ was determined and analyzed by the commonly used “symmetry finder” and “Bilbao Crystallographic Server” tools. The DMI parameter was calculated by the 4 × 1 supercell method proposed by H. Yang et al.^[^ ^53^ ^]^ First, the DMI constant *D* was calculated using the equation $D=3\sqrt{2d}/\left(N_{F}a^{2}\right)$, where *N*_F_ is the number of atomic layer, *a* is the lattice constant, and *d* represents DMI strength. Then the *d* obtained using the formula *d*  =  (*E*_ACW_ − *E*_CW_)/*m*, where *m* is a constant related to the wavelength of cycloid and *E*_ACW_ − *E*_CW_ represents the SOC energy difference Δ*E*_SOC_ between ACW and CW spin configurations, which indicates that Δ*E*_SOC_ is proportional to the DMI constant. Such a method was also applied in the case of bulk systems and interfaces.^[^ ^54^, ^55^ ^]^

### Simulated HAADF‐STEM Images and Micromagnetic Simulations

The simulated HAADF‐STEM images were obtained by using Dr. Probe software.^[^ ^56^ ^]^ Micromagnetic and L‐TEM contrast simulations based on the Landau–Lifshitz–Gilbert (LLG) equation were executed using the MuMax^3^ software.^[^ ^57^ ^]^ Detailed parameters information is noted in the Supporting Information Notes.

## Conflict of Interest

The authors declare no conflict of interest.

## Author Contributions

C.L. and J.J. contributed equally to this work. C.L. conceived the idea and designed the experiments. C.Z. and X.Z. supervised the project. C.Z. and H.Z. performed the crystal growth, characterization, and magnetization measurements. C.L. prepared the TEM samples and collected the atomic resolution HAADF‐STEM images. C.L. and C.Z. performed the L‐TEM experiments under the supervision of X.Z. J.J. conducted the first‐principles calculations. Q.W. conducted the micromagnetic simulations. C.L., C.Z., Z.H., and X.Z. analyzed the data and wrote the manuscript with the help of D.Z., Y.L., Y.M., H.A., X.G., and W.M. All authors contributed to the discussion of the results and improvement of the manuscript.

## Supporting information

Supporting Information

## Data Availability

The data that support the findings of this study are available on request from the corresponding author. The data are not publicly available due to privacy or ethical restrictions.
