# Supplementary material for: Controllable Skyrmionic Phase Transition between Néel Skyrmions and Bloch Skyrmionic Bubbles in van der Waals Ferromagnet Fe3‐δGeTe2
Source: Adv Sci (Weinh). 2023 Jul 28;10(27):2303443. doi: 10.1002/advs.202303443 (PMC10520623; doi:10.1002/advs.202303443)
Supplement: Supplementary file 1 — Supporting Information [file ADVS-10-2303443-s001.pdf]

ADVANCED SCIENCE
Open Access

Supporting Information

for *Adv. Sci.*, DOI 10.1002/advs.202303443

Controllable Skyrmionic Phase Transition between Néel Skyrmions and Bloch Skyrmionic Bubbles in van der Waals Ferromagnet $Fe_{3-\delta}GeTe_2$

Chen Liu, Jiawei Jiang, Chenhui Zhang, Qingping Wang, Huai Zhang, Dongxing Zheng, Yan Li, Yinchang Ma, Hanin Algaidi, Xingsen Gao, Zhipeng Hou*, Wenbo Mi, Jun-ming Liu, Ziqiang Qiu and Xixiang Zhang**

Supporting Information

Controllable Skyrmionic Phase Transition Between Néel Skyrmions and Bloch Skyrmionic Bubbles in van der Waals Ferromagnet $Fe_{3-\delta}GeTe_2$

Chen Liu, Jiawei Jiang, Chenhui Zhang, Qingping Wang, Huai Zhang, Dongxing Zheng, Yan Li, Yinchang Ma, Hanin Algaidi, Xingsen Gao, Zhipeng Hou*, Wenbo Mi, Jun-ming Liu, Ziqiang Qiu and Xi-Xiang Zhang**

This file includes:

Note S1. STEM images simulation.
Note S2. Micromagnetic simulations.
Figure S1. Energy-dispersive X-ray spectrum of $Fe_{3-\delta}GeTe_2$ with different Fe concentrations.
Figure S2. The T_C of $Fe_{3-\delta}GeTe_2$ with various Fe content.
Figure S3. The HAADF-STEM images of [001]-oriented $Fe_{3-\delta}GeTe_2$ lamellas.
Figure S4. The full-size HAADF-STEM images of $Fe_{3-\delta}GeTe_2$ lamellas.
Figure S5. The simulated STEM images of $Fe_{3-\delta}GeTe_2$ lamellas.
Figure S6. EELS line scan results of the h-BN/ $Fe_{3-\delta}GeTe_2$ heterostructures.
Figure S7. The corresponding under-focused L-TEM images of (a) $Fe_{2.69}GeTe_2$ and (b) $Fe_{2.74}GeTe_2$ without tilting.
Figure S8. The corresponding under-focused L-TEM images of $Fe_{2.74}GeTe_2$ under zero tilt condition.
Figure S9. The simulated L-TEM contrasts of several main magnetic structures under different tilting conditions.

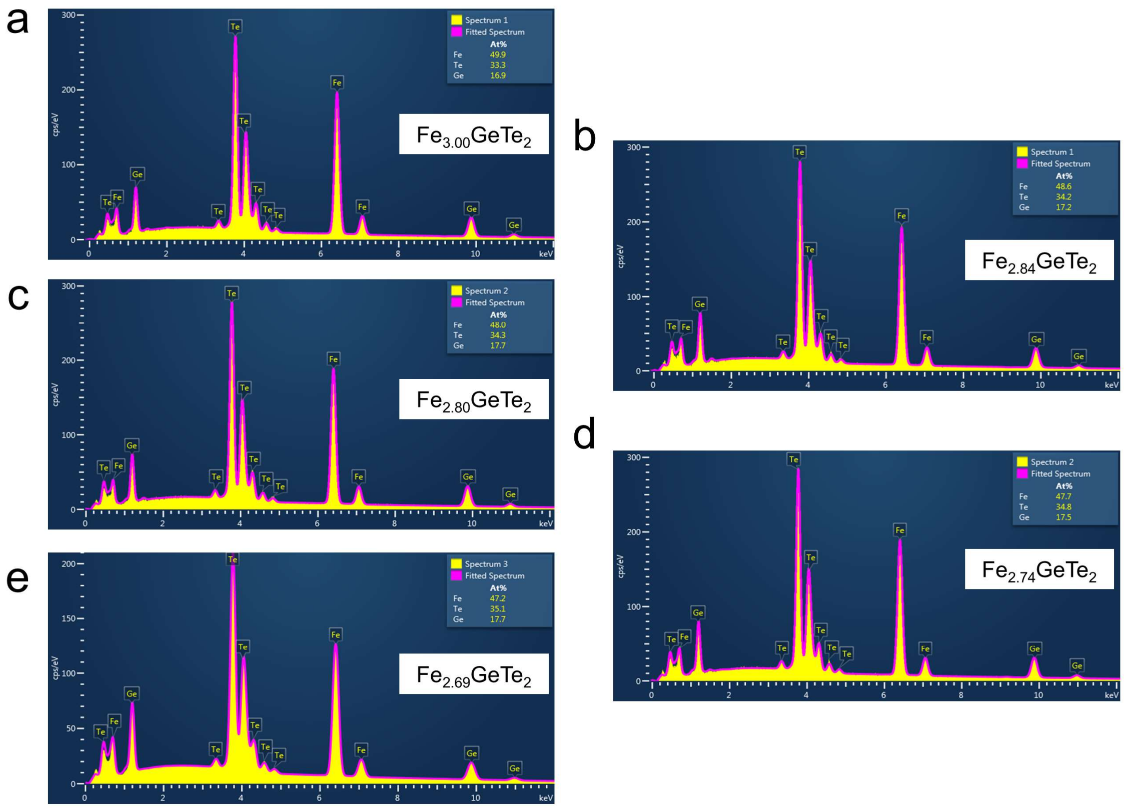

Figure S1. Energy-dispersive X-ray spectrum of $Fe_{3-\delta}GeTe_2$ with different Fe concentrations. The Fe ratio is determined with respect to Te.

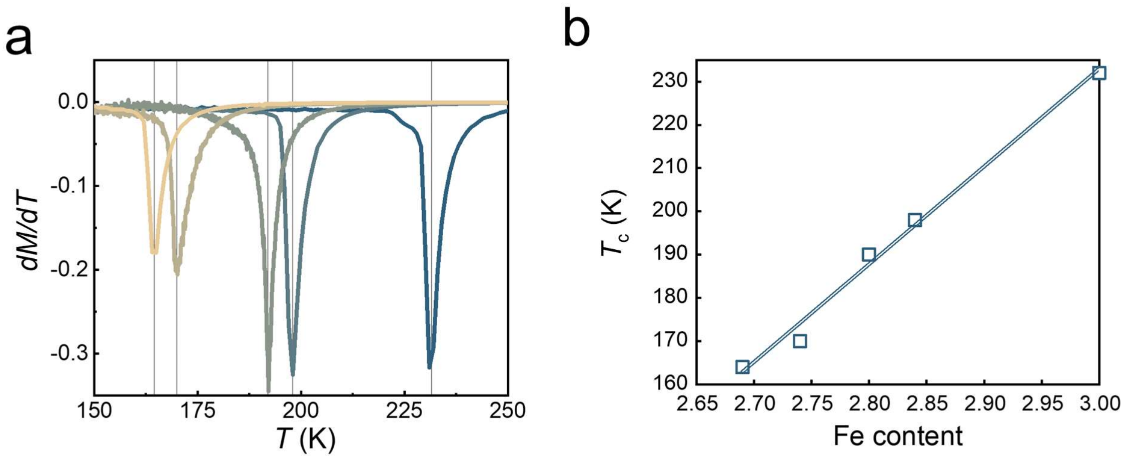

Figure S2. The T_C of $Fe_{3-\delta}GeTe_2$ with various Fe content. (a) The derivative of the FC $M(T)$ curves (Figure1c). (b). The T_C as a function of Fe content.

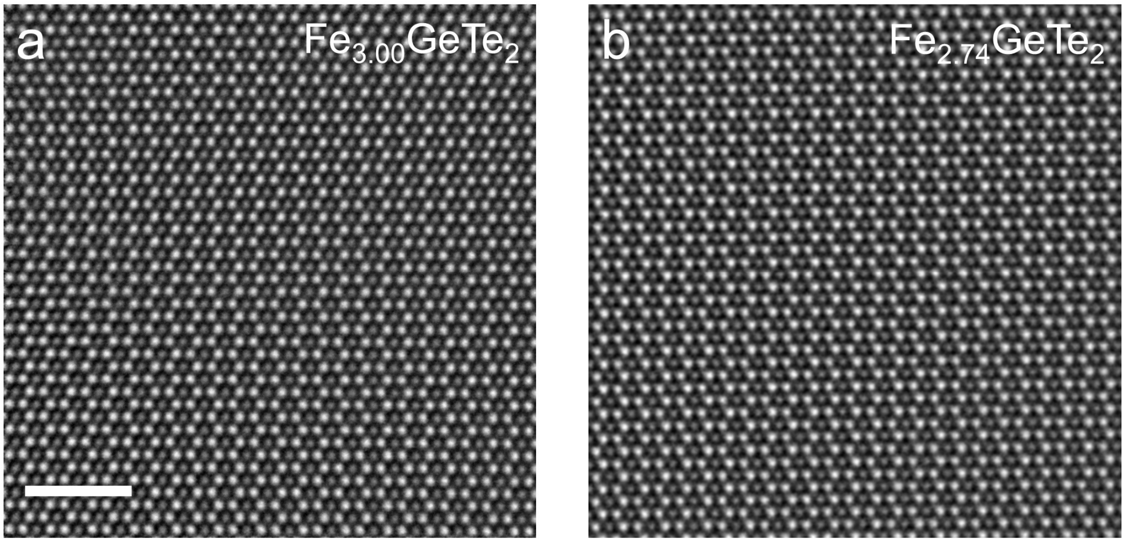

Figure S3. The HAADF-STEM images of [001]-oriented $Fe_{3-\delta}GeTe_2$ lamellas. (a) $Fe_{3.00}GeTe_2$. (b) $Fe_{2.74}GeTe_2$. Scale bar: 2 nm.

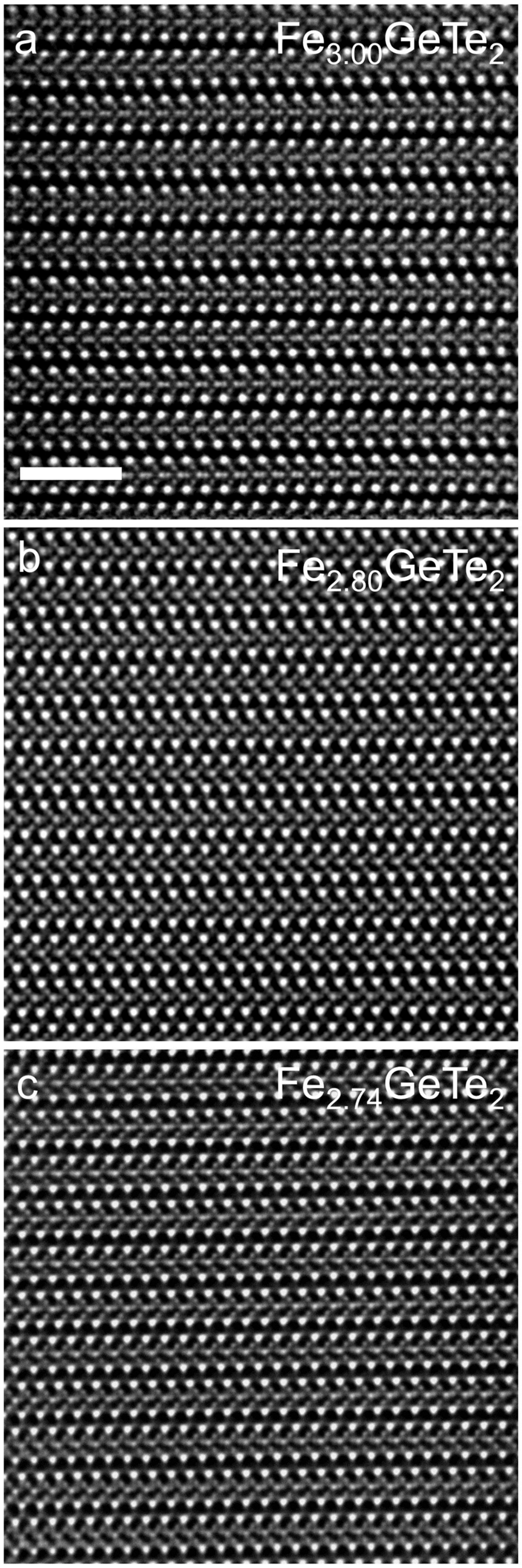

Figure S4. The full-size HAADF-STEM images of $Fe_{3-\delta}GeTe_2$ lamellas. (a) $Fe_{2.74}GeTe_2$, (b)$Fe_{2.80}GeTe_2$ and (c) $Fe_{3.00}GeTe_2$ respectively. Scale bar: 2 nm.

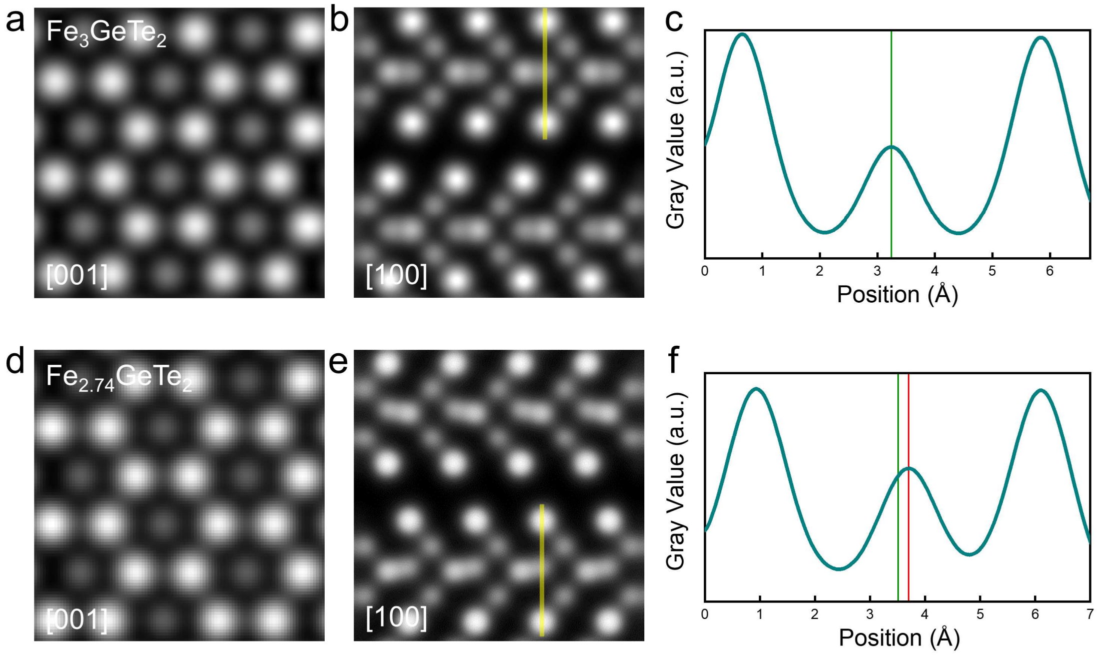

Figure S5. The simulated STEM images of $Fe_{3-\delta}GeTe_2$ lamellas. (a), (b) The simulated STEM images of Fe_3GeTe_2 along the [001] and [100] orientation, respectively. (c) The line profile of the STEM intensity shown in (b). (d), (e) The simulated STEM images of $Fe_{2.74}GeTe_2$ along the [001] and [100] orientation, respectively. (f) The line profile of the STEM intensity shown in (e). The green line represents the central position between two Te atoms and the red line represents the center of the Fe(2) atom. The distance between the red and green lines indicates the Fe(2) atom displacement Δd along the c-axis from the central position.

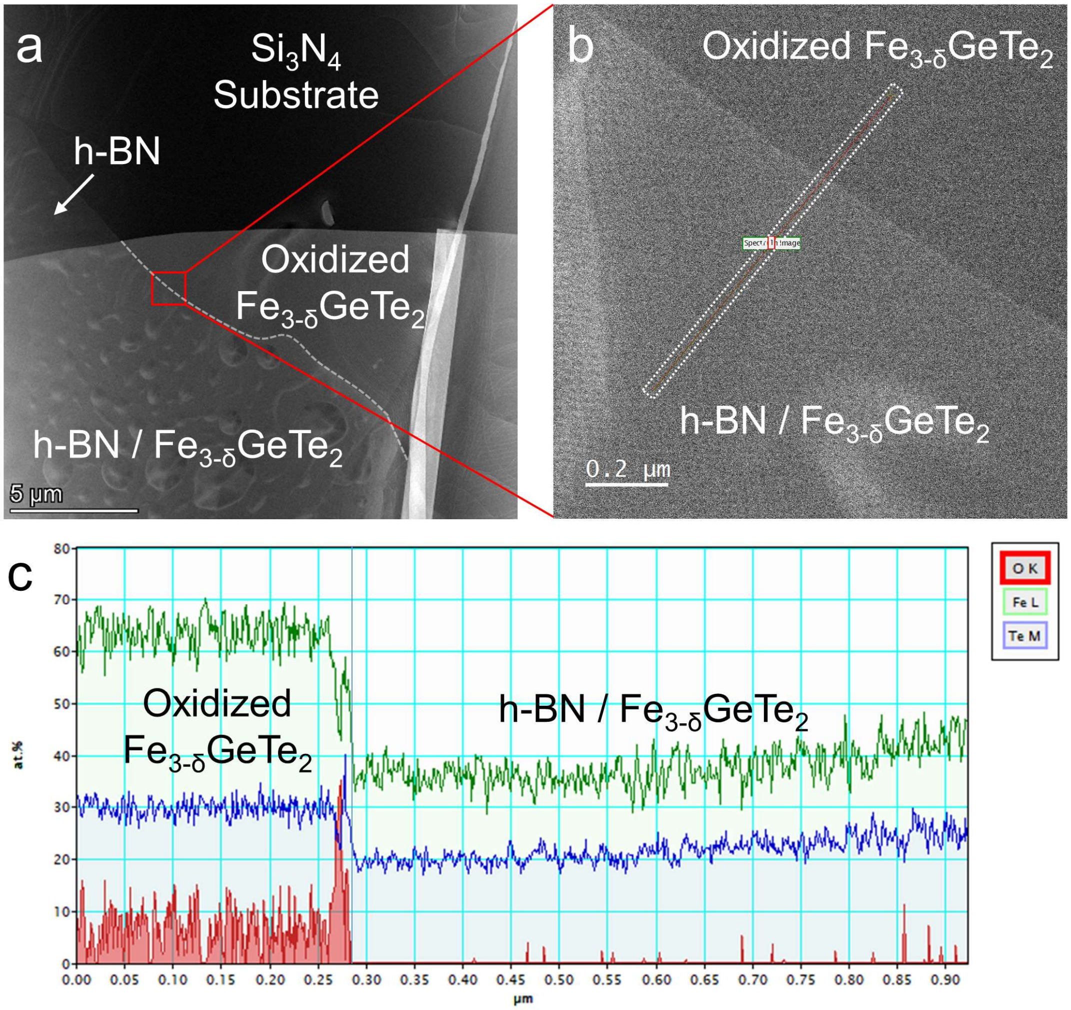

Figure S6. EELS line scan results of the h-BN/ $Fe_{3-\delta}GeTe_2$ heterostructures. (a) HAADF-STEM images of h-BN/ $Fe_{3-\delta}GeTe_2$ heterostructures. The Grey dashed line means the dividing line between h-BN/ $Fe_{3-\delta}GeTe_2$ and oxidized $Fe_{3-\delta}GeTe_2$) regions. (b) The magnified image of the red rectangular area in (a). The red line in the white dash box represents the EELS line scan. (c) The atomic ratio of O, Fe, and Te vs. distance derived from the corresponding EELS line scan in (b).

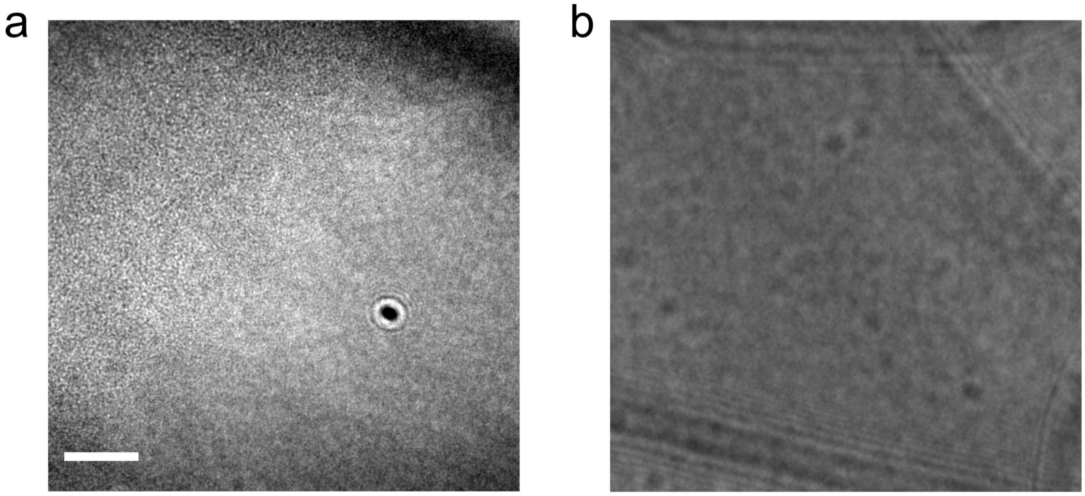

Figure S7. The corresponding under-focused L-TEM images of (a) $Fe_{2.69}GeTe_2$ and (b) $Fe_{2.74}GeTe_2$ without tilting. The defocused value is 3 mm. Scale bar: 1 μm.

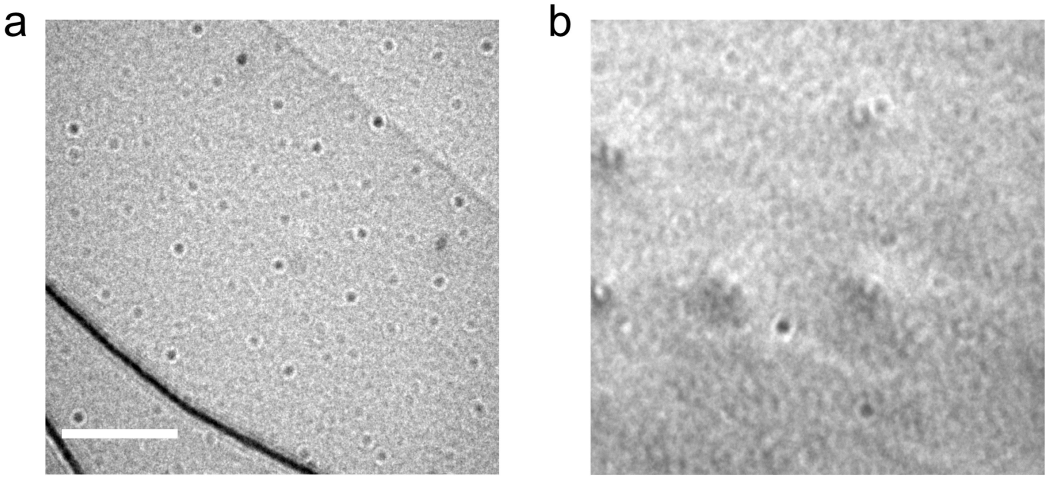

Figure S8. The corresponding under-focused L-TEM images of $Fe_{2.74}GeTe_2$ under zero tilt condition. (a) The L-TEM images of $Fe_{2.74}GeTe_2$ nanoflakes with 44 nm thickness correspond with Figure 4a. (b) The L-TEM images of 70 nm thick $Fe_{2.74}GeTe_2$ nanoflakes correspond with Figure 4b. The defocused value is 3 mm. Scale bar: 1 μm.

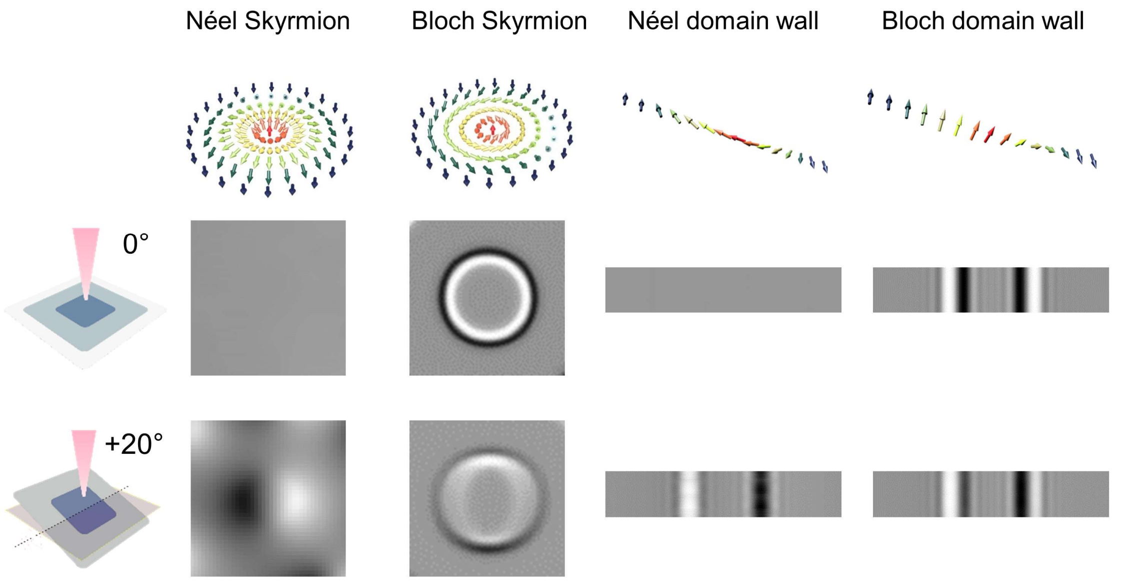

Figure S9. The simulated L-TEM contrasts of several main magnetic structures under different tilting conditions.

Supplementary Note.

1. STEM images simulation.

The STEM simulations were carried out by using Dr. Probe software.[1] The microscope parameters were kept as consistent as possible with the experiment. The parameters were as follows: accelerating voltage 300 kV, alpha 24 mrad, defocus value -5 nm and the HAADF range 70-120 mrad. The thickness of $Fe_{3-\delta}GeTe_2$ lamellas was set as 50 nm. The $Fe_{3-\delta}GeTe_2$ structure parameters were obtained from the previously reported standard Fe_3GeTe_2 structure[2] and the calculation results.

2. Micromagnetic simulations.

Micromagnetic simulations were carried out with Mumax3 software.[3] The Hamiltonian of the $Fe_{3-\delta}GeTe_2$ material system can be expressed as

$$H = E_{tot} = \int_V (E_{ex} + E_D + E_a + E_d + E_Z)\, d\boldsymbol{r}$$
$$= \int_V \left[A|\nabla \boldsymbol{m}|^2 + D\boldsymbol{m} \cdot (\nabla \times \boldsymbol{m}) + K_u(\boldsymbol{u} \cdot \boldsymbol{m})^2 - \frac{1}{2} M_s \boldsymbol{B_d} \boldsymbol{m} - B\boldsymbol{m} \cdot \hat{e}_z \right] d\boldsymbol{r}, \qquad (\mathbf{1})$$

where E_{ex}, E_D, E_a, E_d, and E_Z represent Heisenberg exchange energy, DMI energy, uniaxial anisotropy energy, demagnetization energy and Zeeman energy terms, respectively. Here, $\boldsymbol{m}$ represents a normalized spin and $\boldsymbol{u}$ is a unit vector of magnetic anisotropy. A, D, K_u and M_s are the exchange interaction constant, DMI coefficient, magneto-crystalline anisotropy constant and saturation magnetization, respectively. A $1.2 \times 1.2 \times t$ μm^3 square system with periodic boundary conditions was established as the simulated $Fe_{3-\delta}GeTe_2$ nanoflake system, in which t represents the sample thickness. We set t to 50, 100, 150 and 200 nm, respectively. To simulate the field cooling procedure, an external out-of-plane magnetic field of 150 mT was applied. The $M_s = 4.0\times10^5$ A·m^{-1} and $A = 5\times10^{-12}$ J·m^{-1} were used for simulations, which are cited from previous studies.[4-6] The $D = 0.6\times10^{-3}$ J·m^{-2} was chosen and K_u was varied in the range of 0.5-2.0 J·m^{-3}. The dynamic behavior of magnetic moments was determined by the LLG equation.

The Gilbert damping constant $\alpha = 0.3$ was set to achieve the equilibrium states. The mesh size was set to 4×4 nm^2 in the *xy* plane and 10 nm along the z direction. The corresponding L-TEM contrasts were simulated by using the MALTS code.[7] And the simulated L-TEM images of several typical spin textures under different tilt conditions are listed in Figure S9.

Reference

[1] J. Barthel, *Ultramicroscopy* **2018**, 193, 1.
[2] (Eds: P. Villars, K. Cenzual), Springer-Verlag Berlin Heidelberg & Material Phases Data System (MPDS), Switzerland & National Institute for Materials Science (NIMS), Japan.
[3] A. Vansteenkiste, J. Leliaert, M. Dvornik, M. Helsen, F. Garcia-Sanchez, B. Van Waeyenberge, *AIP Advances* **2014**, 4, 107133.
[4] B. Ding, Z. Li, G. Xu, H. Li, Z. Hou, E. Liu, X. Xi, F. Xu, Y. Yao, W. Wang, *Nano Lett* **2020**, 20, 868.
[5] T.-E. Park, L. Peng, J. Liang, A. Hallal, F. S. Yasin, X. Zhang, K. M. Song, S. J. Kim, K. Kim, M. Weigand, G. Schütz, S. Finizio, J. Raabe, K. Garcia, J. Xia, Y. Zhou, M. Ezawa, X. Liu, J. Chang, H. C. Koo, Y. D. Kim, M. Chshiev, A. Fert, H. Yang, X. Yu, S. Woo, *Physical Review B* **2021**, 103, 104410.
[6] A. Chakraborty, A. K. Srivastava, A. K. Sharma, A. K. Gopi, K. Mohseni, A. Ernst, H. Deniz, B. K. Hazra, S. Das, P. Sessi, I. Kostanovskiy, T. Ma, H. L. Meyerheim, S. S. P. Parkin, *Advanced Materials* **2022**, 34, 2108637.
[7] S. K. Walton, K. Zeissler, W. R. Branford, S. Felton, *IEEE Transactions on Magnetics* **2013**, 49, 4795.
